# Enhancing Production and Cytotoxic Activity of Polymeric Soluble FasL-Based Chimeric Proteins by Concomitant Expression of Soluble FasL

**DOI:** 10.1371/journal.pone.0073375

**Published:** 2013-08-26

**Authors:** Aurore Morello, Sophie Daburon, Michel Castroviejo, Jean-François Moreau, Julie Dechanet-Merville, Jean-Luc Taupin

**Affiliations:** 1 CNRS UMR 5164 CIRID, Université Bordeaux Segalen, Bordeaux, France; 2 CNRS UMR 5234, Université Bordeaux Segalen, Bordeaux, France; 3 Laboratoire d’Immunologie et immunogénétique, CHU de Bordeaux, Bordeaux, France; IISER-TVM, India

## Abstract

Membrane FasL is the natural trigger of Fas-mediated apoptosis. A soluble homotrimeric counterpart (sFasL) also exists which is very weakly active, and needs oligomerization beyond its trimeric state to induce apoptosis. We recently generated a soluble FasL chimera by fusing the immunoglobulin-like domain of the leukemia inhibitory factor receptor gp190 to the extracellular region of human FasL, which enabled spontaneous dodecameric homotypic polymerization of FasL. This polymeric soluble human FasL (pFasL) displayed anti-tumoral activity *in vitro* and *in vivo* without systemic cytotoxicity in mouse. In the present work, we focused on the improvement of pFasL, with two complementary objectives. First, we developed more complex pFasL-based chimeras that contained a cell-targeting module. Secondly, we attempted to improve the production and/or the specific activity of pFasL and of the cell-targeting chimeras. We designed two chimeras by fusing to pFasL the extracellular portions of the HLA-A2 molecule or of a human gamma-delta TCR, and analyzed the consequences of co-expressing these molecules or pFasL together with sFasL on their heterotopic cell production. This strategy significantly enhanced the production of pFasL and of the two chimeras, as well as the cytotoxic activity of the two chimeras but not of pFasL. These results provide the proof of concept for an optimization of FasL-based chimeric proteins for a therapeutic use.

## Introduction

FasL (CD95L) is a type II homotrimeric transmembrane protein of the TNF family [1]. FasL is expressed on activated T lymphocytes and natural killer cells, as a mean to eliminate transformed or infected cells expressing the transmembrane receptor Fas (CD95/APO-1) [2]. Cleavage of membrane-bound FasL by metalloproteases [3,4] generates homotrimeric soluble FasL (sFasL), which is at least 1000-fold less apoptotic, and competes with membrane FasL for cell killing [5–8]. Interestingly, upon cross-linking with antibodies, sFasL recovers its pro-apoptotic activity, and a FasL hexamer appears as the smallest functional form [9].

The triggering of Fas initially appeared as a promising approach to treat cancer but an agonistic anti-Fas antibody led to fulminant lethal hepatitis upon injection in mice, precluding the use of Fas inducers for a therapeutic purpose in human [10]. A humanized anti-Fas antibody did not display such toxicity but frequently triggered anti-idiotype sensitization in monkeys [11]. Besides this approach, numerous research teams have designed sFasL-derived agonists devoid of the toxicity of the anti-Fas antibodies and with at least comparable apoptotic activity [reviewed in references 12,13, using either spontaneously polymeric (i.e. at least hexameric) or oligomeric (i.e. smaller than the hexamer) sFasL-based fusion proteins. These chimeras contained or not, N-terminally to sFasL, a targeting module able to recognize the target cell to be destroyed or to be recognized by a carrier cell, which will then present FasL to the target cell, thereby mimicking transmembrane FasL. Representative examples were the hexameric MegaFasL [9] which associates the collagen domain of adiponectin (ACRP30) to sFasL, the streptavidin-FasL (SA-FasL) [14], and the CTLA-4-FasL [15,16] chimeras, which respectively bind to biotin-labeled or to CD80-expressing cells. An alternative strategy consisted in fusing to sFasL a single chain variable fragment from a monoclonal antibody, as for sc40-FasL [17] and scfvRit-FasL [18], to target fibroblasts and B-cell leukemia cells through the Fibroblast Activation Protein and CD20, respectively. *In vivo* assays in rodent models have demonstrated the validity of these strategies for immunotherapy, as they helped controlling allogenic immune reactions, tumor progression or experimental arthritis [19], alone or in combination with chemotherapeutic agents [reviewed in 12,13]. MegaFasL is currently being used in human glioma [ref [20]. and NIH clinical trial NCT004437736)].

We recently developed a new form of functional polymeric human sFasL (pFasL) which spontaneously occurs in cell culture supernatants as cytotoxic hexamers and dodecamers. It is a fusion protein between human sFasL and an 81 amino acids subdomain of the human Leukemia Inhibitory Factor (LIF) receptor chain gp190 having a spontaneous propensity to self-associate. It displays a higher cytotoxic activity than the commercially available antibody-crosslinked Flag-tagged sFasL (sfFasL), and is able to delay tumor growth in a murine model of transplanted human tumor cells [21]. To further improve this recombinant death inducer, we focused on two complementary objectives. The first one was to develop pFasL-derived chimeras containing a cell-targeting module in order to selectively enhance the activity of pFasL. The second one was to improve the cell production and/or the specific activity of pFasL itself and of such chimeric products. Indeed, we followed the intuitive reasoning that compacting into a polymeric complex, monomers each consisting of successive protein modules, may induce increasing structural constraints that will finally be detrimental to the chimera, either for its intracellular production or secretion, or for its targeting specificity and/or cytotoxic activity.

We thus designed two chimeras, in which a Flag-tagged form of pFasL (pfFasL) was respectively C-terminally linked to a beta-2 microglobulin/HLA-A*02: 01 fusion molecule (HLA-pfFasL) or to the extracellular portions of a Vγ4Vδ5 gamma-delta TCR (TCR-pfFasL) able to recognize the cellular Endothelial Protein C receptor (EPCR). These examples of targeting modules were selected as possible strategies to eliminate by Fas-mediated apoptosis respectively HLA-alloreactive T-lymphocytes in a transplantation setting, or carcinoma cells as EPCR is a stress self antigen over-expressed in various cancer cell types and recognized by the Vγ4Vδ5 TCR [22]. To verify our hypothesis, we co-expressed with the cDNA encoding pFasL or the chimera, the one encoding the very weakly apoptotic sFasL, expecting it to be incorporated into the secreted chimeric polymer and therefore able to improve overall structure of the complex while maintaining its activity. In the present report, we describe the biochemical and functional characteristics of the complexes generated.

## Materials and Methods

### Cell lines, chemicals and antibodies

The human Fc receptor CD32 transfected mouse fibroblastic L-cells [23], the simian epithelial COS-7 [24] and the human epithelial HEK 293T [25] cell lines were maintained in culture with DMEM (Invitrogen Gibco, Fisher Scientific, Illkirch, France). The human T-lymphoma Jurkat cells [26] were cultured in RPMI 1640 (Invitrogen Gibco). Culture media were supplemented with 8% heat-inactivated FCS (GeHealthcare, Buckinghamshire, UK) and 2 mM L-glutamine (Sigma, Saint-Louis, USA). The PE-labelled anti-CD32 and anti-mouse IgG mAbs used for cell staining were from Immunotech Beckman Coulter (Marseille, France). The anti-mouse Fas (clone JO-2) and the anti-human FasL (clone G247-4) mAbs were from BD Biosciences (Pont de Claix, France). The purified anti-Flag (clone M2), anti-β2 microglobulin (clone B2M-01) and anti-CD32 (clone AT10) mAbs were from Sigma, Pierce technology (Rockford, USA) and Abcam, (Cambridge, USA), respectively. The mouse anti-human FasL clones 10F2 (neutralizing) and 14C2 (non-neutralizing) mAbs were home-made [27]. The IgG1 and IgG2 isotype negative control anti-LIF clones 1F10 and 7D2 were also home-made [28]. The remaining chemical reagents were purchased from Sigma unless otherwise specified.

### Plasmid constructs

All the constructs were subcloned into the 5370 bp pEDr mammalian expression vector [29]. The soluble FasL (sFasL) and the soluble polymeric FasL (pFasL) constructs were previously described [21]. Regarding the TCR-pFasL, two constructs were generated by fusing the extracellular regions of the γ4 TCR chain (aa 20 to 295) or of the δ5 TCR chain (aa 27 to 272) to the pFasL coding sequence as follows. The portion encoding the extracellular domain of the γ4 TCR chain or of the δ5 TCR chain [22] were obtained by PCR using 5’-AATCTAGACAGCAAGTTAAGCAAAATTC-3’ and 5’-AAACTAGTTGTGAGGGACATCATGTTC-3’ primers for the δ5 chain or 5’ AATCTAGAAACTTGGAAGGGAGAACG- 3’ and 5’- AAACTAGTCAGGAGGAGGTACATGTA-3’ primers for the γ4 chain. The PCR fragments were digested by XbaI and SpeI enzymes and ligated into the pEDR-pFasL vector into the SpeI cloning site. For the HLA-pFasL construct, the extracellular domain of the HLA-A*02: 01 sequence fused 3’-terminally to the beta2-microglobulin whole coding sequence kindly provided by Dr Jar-How Lee (One Lambda, Canoga Park, CA), was subcloned into the pFasL plasmid as follows. The fragment encompassing the signal peptide and extracellular portion of this chimera (aa 1 to 386) were isolated by PCR using 5’-AGATCTAAGGAGATATAGATATGTCTCGCTCCGTGGCC-3’ and 5’- ACTAGTACTACCGGCACCTCCCAGGGGAGGGGCTTGGG-3’ primers. A 15 bp linker (GGAGGTGCCGGTAGT) was added to the 3’ overhang by PCR. The whole PCR fragment was ligated into the pEDr-pFasL vector between the BglII and SpeI cloning sites. A 21-bp Flag tag sequence containing 5’ XbaI and 3’ SpeI overhangs was added between the TCR or HLA modules and the pFasL portion by direct ligation into a SpeI site, generating the TCR-pfFasL constructs. The pfFasL and sfFasL constructs were obtained similarly. All the constructs were verified by sequencing (Beckman Coulter Genomics, Takeley, UK). The final plasmids encoding sFasL, sfFasL, pfFasL, TCR-pfFasL and HLA-pfFasL displayed a nucleotide length in the range of 6000, 6000, 6300, 7100 and 7400 base pairs. For the transfection experiments using mixed plasmids, the percentage of added sFasL plasmid was determined on a molar basis.

### Production of the soluble chimeras by calcium phosphate transient transfection

The human sFasL, sfFasL, pfFasL and HLA-pfFasL recombinant proteins were produced by transient expression in COS-7 cells whereas TCR-pfFasL was expressed in HEK 293T cells as higher amounts were produced in this cell line, according to the protocol optimized by Jordan et al [30]. One day before transfection, 1.5.10^6^ cells were seeded in a 10 cm Petri dish in complete medium. The medium was replaced 3 to 4 hours prior to transfection. The plasmid DNA (7.6 pmol, corresponding to 30 µg in the case the sfFasL encoding plasmid) was diluted to the indicated concentration with Ultrapure (Sigma) water and 2 M calcium chloride (70 µl/dish) to a final volume of 0.5 ml. After adding one volume of 2X HBS buffer (pH 7.05; 1.5 mM Na_2_HPO_4_, 55 mM HEPES, 274 mM NaCl) the mix was allowed to precipitate for 10 min at room temperature and added dropwise onto the plated cells. The supernatants containing targeted soluble chimeras were collected 4 days after the transfection and centrifuged 20 min at 4000 rpm at 4°C and the pelleted debris were removed. For the TCR-pfFasL, the plasmids containing the TCR γ4 chain and the TCR δ5 chain were co-transfected in equal amounts (w/w).

### Protein quantification

The concentration of the chimeras was quantified in cell culture using specific sandwich ELISA assays. The anti-FasL 14C2 or the anti-Flag mAbs were pre-coated overnight onto 96 well ELISA plates (Maxisorp Nunc, Thermo Scientific, Rochester, USA) respectively at 1 µg or 0.25 µg/well in hydrogenocarbonate coating buffer (pH = 9.6). The plate was washed 3 times with PBS containing 0.05% Tween 20 and saturated with PBS containing 1% BSA. Known quantities of untagged pFasL or sfFasL were used as standards, respectively. After 2-h incubation with 100 µl/well of the chimeras to be measured, the plate was washed and incubated 1 h with biotinylated anti-human FasL mAb 10F2 at 0.1 µg/well in 100 µL diluted in PBS with 1% BSA. After 3 washes, the plate was incubated for 1 h with peroxidase-labelled streptavidin (GEHealthcare) diluted 1/2000 in PBS with 1% BSA. After 1 h incubation and a final wash step, the tetramethylbenzidine substrate (60 µg/ml in pH 5.5 citrate buffer) was added (100 µl/well). The reaction was stopped after 15 min with 1 M sulfuric acid (50 µl/well) and the plate was read at 450 nm on a spectrophotometer.

### Cytotoxicity assays

The cytotoxic activity of the chimeras was evaluated on Jurkat cells using the MTT viability assay. Cells (3x10^4^/well) were seeded in flat-bottomed 96 well-plates and incubated overnight with the chimeras in a final volume of 100 µl in duplicates. Then, cells were incubated for 4 h at 37°C with the tetrazolium salt [3-(4,5-dimethyl thiazol-2yl)]-2,5-diphenyl tetrazolium bromide (Sigma), 15 µl/well at 5 mg/ml in PBS. After addition of 105 µl/well of 5% formic acid in isopropanol to solubilize the formazan precipitate, optical density was measured at 570 nm. The percentage of specific cytotoxicity of the chimera on the cells was then calculated as follows: 100-[(experimental absorbance - background absorbance Jurkat cells alone)/(control absorbance- background absorbance)] x 100.

The enhancing effect of the chimera-targeting module was analyzed on L-cells stably expressing human CD32 using a propidium iodide cytotoxicity assay as follows. The HLA-pfFasL chimera at the indicated concentration was incubated during 1 h at room temperature with an anti-β2 microglobulin mAb at 0.12 µg/ml, the anti-Flag mAb at 0.04 µg/ml or an IgG1 isotype-matched negative control at 0.12 µg/ml, to a final volume of 50 µl. These concentrations provided the optimal cross-linking effect in dose–response experiments with the L-cells. Then, 2.x10^4^ /well L-cells were added to a final volume of 0.1 ml. Regarding the blocking experiments, L-cells were pre-incubated 30 min at room temperature with anti-CD32 (clone AT10) or with anti-FasL (clone 10F2) blocking mAbs at 5 µg/ml, respectively. The plates were incubated at 37°C during 36 h. Cells and apoptotic bodies were centrifuged 10 min at 4000 rpm and resuspended with propidium iodide solution (50 µg/ml) (Sigma) diluted in hypotonic solution (0.1% trisodium citrate, 0.1% triton X100) and the percentage of cells in sub-G1 was analyzed by flow cytometry (Fortessa, BD Biosciences).

### Immunoprecipitation and immunoblot experiments

Chimera immunoprecipitations were performed using Pansorbin® from *S. aureus* cells (EMD Millipore, Darmstadt, Germany). Pansorbin® (4 µl/condition), pre-saturated with PBS containing 3% BSA, was incubated overnight at 4°C with 3 µg of purified anti-Flag or anti-FasL 10F2 mAbs in a total volume of 1 ml. The excess of unbound mAb was removed by adding 1 ml of washing buffer (25 mM HEPES pH 7.4, 40 mM Na _4_P_2_O_7_, 100 mM NaF, 40 mM Na _3_VO_4_, protease cocktail inhibitor, Triton 0.5%), followed by centrifugation (5500 rpm, 5 min, 4°C). A fixed concentration of the chimera quantitated with the Flag/FasL ELISA was then added to the pellet to a final volume of 0.7 ml. After 4 h incubation at 4°C, the pellet was centrifuged and washed 4 times with the washing buffer. The proteins were released by heating (95°C, 5 min) in reducing loading buffer before separation by SDS-PAGE.

For the immunoblot experiments, either supernatant or immunoprecipitated proteins were electrophoretically separated by SDS-PAGE on 10 or 15% gels in reducing conditions, and transferred onto nitrocellulose membrane (Biotrace NT, VWR, Fontenay-sous-bois, France) by semi-dry transfer. The membranes were stained with Ponceau red and saturated with 2.5% BSA in TBST buffer (192 mM Glycine, 25 mM Tris, 0.1% SDS, 0.05% Tween 20, pH 7.9). Immunoblots were performed with the mouse anti-human FasL G247-4 antibody at 2.5 µg/ml in TBST and with an IRDye® 800CW labelled anti-mouse IgG antibody (LICOR® ScienceTech, Courtaboeuf, France) at a 1/10000 dilution in TBST. Then, the luminescence signal were visualized and quantified by densitometry with the Odyssey® Infrared Imaging system (LICOR®).

### Statistical analysis

Statistics were calculated with the t test using Statview (SAS Institute Corporation, Version 5.0, Cary, NC) software.

## Results

### Description of the FasL-based proteins

**Figure 1:**
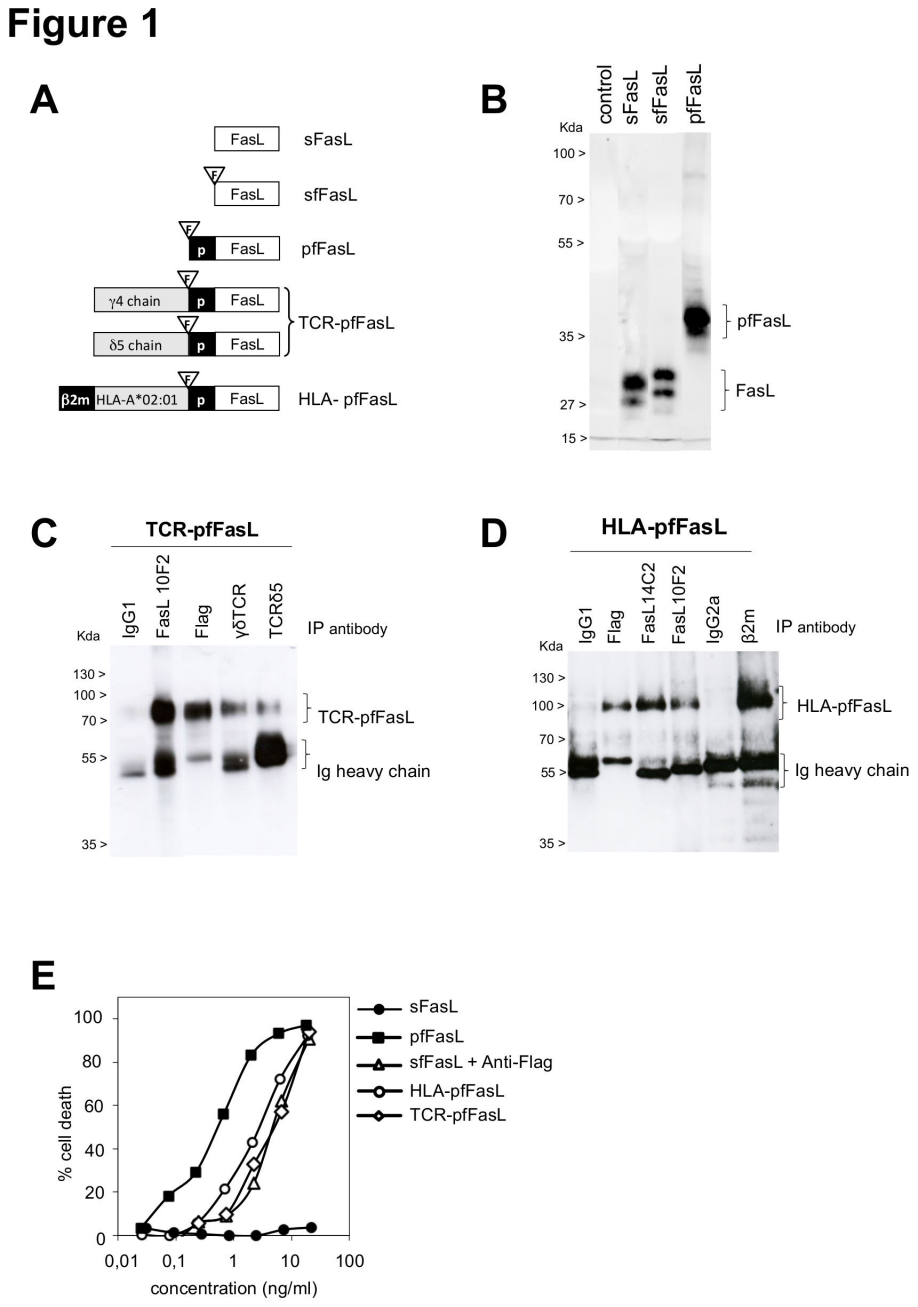
Description and characterization of the chimeric human FasL-derived constructs Panel A: Schematic representation of soluble FasL (sFasL), Flag-tagged sFasL (sfFasL), polymeric Flag-tagged soluble FasL (pfFasL), polymeric TCR γ4 and δ5 Flag-tagged soluble FasL generating the TCR-pfFasL upon cotransfection, and beta2-microglobulin-fused HLA-A*02: 01 Flag-tagged soluble FasL (HLA-pfFasL). The f and p symbols represent the flag epitope and the LIF receptor-derived domain triggering the polymerisation of the FasL oligomers, respectively. Panel B: direct immunoblot of the supernatants from COS cells transfected with the empty vector (control) or the FasL constructs sFasL, sfFasL and pfFasL. Panel C: immunoprecipitation of the TCR-pfFasL chimera from transfected HEK cells, using an irrelevant IgG1 antibody, the anti-Flag (clone M2), the anti-FasL (clone 10F2), the anti-TCRγδ (clone IMU-510) or the anti-TCRδ5 (clone 12C7) antibodies. Panel D: immunoprecipitation of the HLA-pfFasL chimera from the supernatant of COS cells, with anti-Flag, anti-FasL or anti-β2microglobulin antibodies. As controls, the same experiment was performed with irrelevant IgG1 and IgG2 antibodies. Panel E: cytotoxic effect of the FasL chimeras. The indicated chimeras, as supernatants from transfected cells and quantitated using the ELISA for FasL, were incubated at the indicated concentrations with Jurkat cells. After 18 h, the MTT cell viability assay was performed. The anti-Flag M2 antibody at 0.5 µg/ml was added to sfFasL to render it cytotoxic.

The 6 FasL-derived recombinant proteins used in the present study are schematically described in Figure 1A. Besides soluble FasL (sFasL) and its Flag-tagged sfFasL counterpart, we also modified pFasL to incorporate the Flag tag, leading to pfFasL. We also generated three constructs associating a cell-targeting module N-terminally to pfFasL, which were the extracellular segments of the HLA-A*02: 01 allele fused to the beta-2 microglobulin coding sequence, and of a Vγ4Vδ5 TCR, leading to the HLA-pfFasL, the γ4-pfFasL and the δ5-pfFasL molecules, respectively.

The recombinant proteins were all secreted as soluble forms in the supernatant of transfected mammalian cells. The TCR being a heterodimeric protein, the TCR-pfFasL protein was produced upon co-transfection of equal amounts of the plasmids encoding γ4-pfFasL and δ5-pfFasL. The low molecular weight sFasL, sfFasL and pfFasL monomers appeared as two distinct forms relating to different levels of glycosylation, as previously reported [31] and as shown in Figure 1B. As expected, the pfFasL, TCR-pfFasL and HLA-pfFasL were polymeric, and after immunoblotting under reducing conditions displayed apparent sizes of 37-40 (Figure 1B), 79 (Figure 1C) and 85 kDa (Figure 1D) respectively. We observed that the larger chimeric proteins HLA-pfFasL and TCR-pfFasL were produced at much lower levels (36±18 ng/ml and 133±46 ng/ml respectively) than sfFasL (17±8.5 µg/ml), whereas pfFasL was secreted at an intermediate level (3.3±2.9 µg/ml). The large HLA-pfFasL and TCR-pfFasL chimeras were cytotoxic with a C50 in the ng/ml range (Figure 1E). The size, production and biological activity characteristics of the FasL proteins are summarized in Table 1. Globally, increasing the complexity and the size of the chimeras deeply and negatively altered the amount of protein secreted in the culture supernatants. Specifically, we observed that sFasL and sfFasL were barely cytotoxic on their own, as they were at least 5000 less active than pfFasL (C50 measured at >3000 vs 0.6+/-0.4 ng/ml, respectively). This was caused by an insufficient degree of polymerization of the soluble forms of FasL, as demonstrated by cross-linking of sfFasL with the anti-Flag antibody which allowed for the recovery of an activity close to that of pfFasL (3+/-1.3 vs 0.6+/-0.4 ng/ml).

**Table 1:** Main characteristics of the FasL-derived proteins used in the present study, in terms of production, size and cytotoxic activity.

**Proteins**	**Molecular weight (monomer, kDa)**	**EC50 (ng/mL)**	**n^1^**
sFasL	27-30	> 3000	5
sfFasL	29-32	> 3000	5
sfFasL+anti-Flag	-	3 +/-1.3	5
pfFasL	37-40	0.6 +/- 0.4	8
TCR-pfFasL	79	3.7 +/- 1.2	12
HLA-pfFasL	85	2.9 +/- 1.3	15

^1^ number of experiments conducted from different transfection supernatants used for the determination of the cytotoxicity EC_50_ values on the Jurkat cell line.

### Enhancement of FasL-derived chimera production in the presence of sFasL

We hypothesised that the concomitant transfection of the plasmid encoding the small sFasL compound could enhance the release of our FasL-derived chimeras into the supernatant. In preliminary experiments, we observed for pfFasL, TCR-pfFasL and HLA-pfFasL, that the amount of recombinant protein produced reached its maximum for the experimental transfection conditions used, i.e. using 30 µg of plasmid (Figure S1), according to previously published results [32].

**Figure 2:**
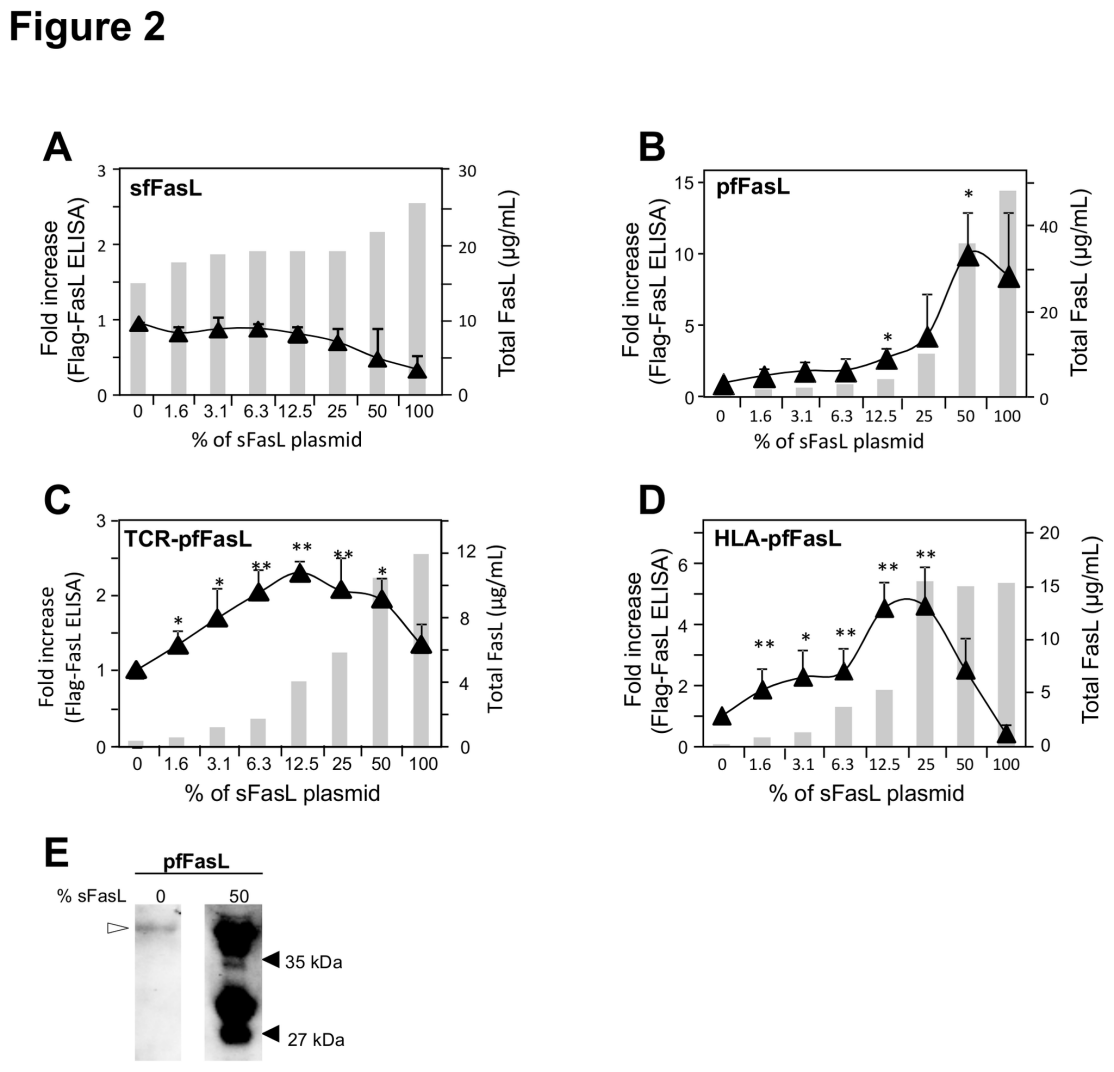
Effect of sFasL on the supernatant production of the Flag-tagged FasL constructs. Panels A to D: An increasing amount expressed in percentage, of the sFasL encoding plasmid, was co-transfected with a fixed amount of the plasmids encoding sfFasL (Panel A), pfFasL (Panel B), TCR-pfFasL (Panel C) and HLA-pfFasL (Panel D). The secreted proteins were quantified in culture supernatants using an ELISA specific for FasL (shaded histograms, right-hand scale) and for Flag-tagged FasL (curves, left-hand scale). For the Flag ELISA, the measured concentrations were normalized according to the condition lacking sFasL. Are presented the mean +/- sd of four independent transfection experiments. * 0.02≤p≤0.05; ** p≤0.02. Panel E : direct anti-FasL immunoblot analysis of identical volumes of the cell culture supernatant containing pfFasL produced alone and with 50% of the sFasL plasmid, after SDS-PAGE separation under reducing conditions.

We then tested the effect on sfFasL and pfFasL production, of the co-transfection of increasing amounts of the sFasL encoding construct together with a fixed amount of these plasmids (Figure 2A and 2B, respectively). Total FasL protein was measured using an ELISA with two antibodies recognizing distinct epitopes of FasL, whereas sfFasL and pfFasL were discriminated from sFasL with an ELISA using anti-Flag and anti-FasL antibodies for the capture and detection steps, respectively. We indeed observed that the sFasL plasmid added at the time of the transfection, dose-dependently increased the amount of, pfFasL produced in the supernatant. In our hands, the optimal molar ratio between both cDNA species was reached with 50% of the sFasL plasmid, leading to a 10-fold rise of the supernatant concentration of pfFasL when compared to pfFasL produced alone (i.e. the 0% condition for sFasL plasmid), while the total amount of FasL-containing proteins increased concomitantly. In contrast, for the sfFasL construct, we were not able to demonstrate any enhancing effect of sFasL on the level of Flag-tagged FasL protein produced and the total amount of FasL containing protein increased only weakly.

The higher molecular weight chimeras TCR-pfFasL and HLA-pfFasL were also examined (Figure 2C and 2D, respectively). A significant enhancing effect of sFasL was obtained on the production of both Flag-tagged constructs, with a maximum for 12.5% and 12.5 to 25% of the amount of the TCR-pfFasL and HLA-pfFasL, respectively. This allowed increasing the amount of the chimeras by 2 and 5 fold above the level reached when they were expressed alone. In addition, as observed for pfFasL, the total amount of FasL containing protein increased with the amount of plasmid transfected, but the quantity of the Flag-tagged chimera produced significantly decreased for the highest amounts of sFasL plasmid. The apparent increase in Flag-tagged protein production in the presence of sFasL, as measured with ELISA, was verified by directly immunoblotting with the anti-FasL antibody the cell culture supernatant obtained at the optimal condition of plasmid ratio. As shown in Figure 2E for pfFasL, we confirmed that the co-transfection strongly increased the production of pfFasL (MW 37-40 kDa) and that sFasL was also produced (MW 27-30 kDa).

### Physical association of sFasL to the pfFasL-derived chimeras

**Figure 3:**
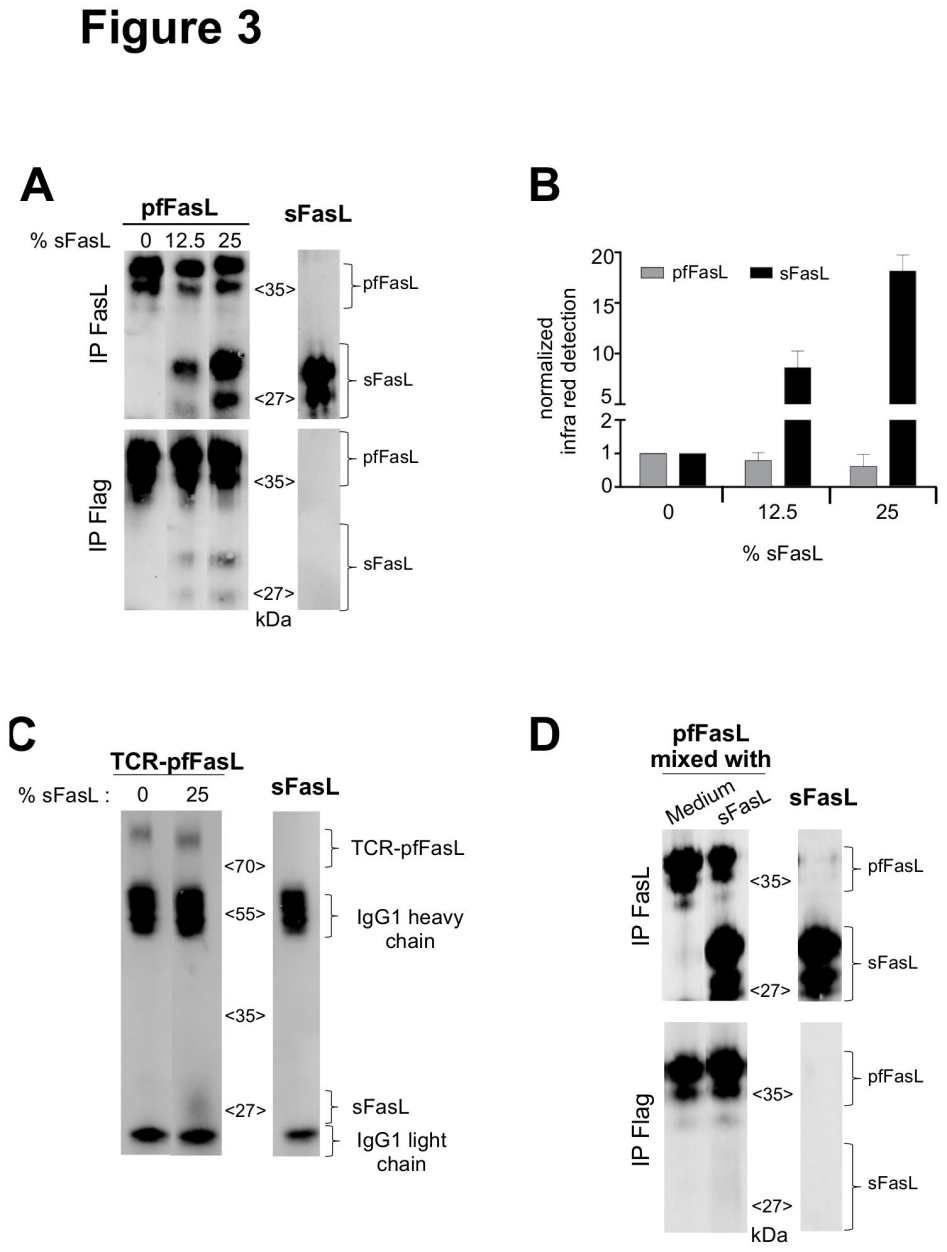
Direct association of sFasL to the pfFasL-containing chimeric proteins during co-expression. Panel A: Identical amounts of pfFasL (1 µg, according to the Flag ELISA) produced in the presence of the indicated ratios of added sFasL plasmid (left panels) was immunoprecipitated with the anti-FasL (upper panel) or anti-Flag (lower panel) antibodies, followed by a SDS-PAGE under reducing conditions and immunoblotting with an anti-FasL antibody. As a control, the same experiment was performed for the sFasL molecule (3 µg according to the FasL ELISA, right panel). Panel B: Densitometric detection and quantification of the pfFasL (grey bars) and the sFasL (black bars) fractions, following transfection of the pfFasL plasmid in the presence of the indicated proportion of the sFasL plasmid. The measures were normalized to the condition lacking sFasL. Mean+/- sd from three experiments. Panel C: The TCR-pfFasL chimera (2 µg, according to an ELISA specific for the TCR-pFasL molecule using anti-TCRδ5 (clone 12C7) and anti-FasL (clone 10F2) as capture and tracing antibodies, respectively), produced in the absence or the presence of the sFasL plasmid at the indicated ratio, was immunoprecipitated with the anti-TCRδ5 antibody, then separated by 10% SDS-PAGE under reducing conditions and revealed by immunoblotting with the anti-FasL antibody. As a control, the immunoprecipitation experiment was performed with 2 µg of sFasL protein. Panel D: COS supernatants containing pfFasL (4 µg/ml according to the Flag ELISA) produced alone, was mixed with culture medium or sFasL (15 µg/ml) produced separately in a total volume of 1 ml, and incubated for 24 h at 37°C. Then the recombinant proteins were immunoprecipitated (left panels) with the anti-FasL (upper panel) or anti-Flag (lower panel) antibodies, followed by a SDS-PAGE under reducing conditions and immunoblotting with an anti-FasL antibody. As a control, the same experiment was performed for the sFasL molecule (15 µg according to the FasL ELISA, right panel).

We then asked whether sFasL, when expressed together with the pfFasL-derived constructs, could physically associate with the polymeric chimera. Firstly, immunoprecipitation experiments were carried out for the pfFasL chimera with anti-FasL or anti-Flag antibodies, followed by immunoblotting with an anti-FasL antibody (Figure 3A). The untagged sFasL produced alone as a control was immunoprecipitated with the anti-FasL but not with the anti-Flag antibody. No sFasL was detected in the anti-FasL immunoprecipitates of the pfFasL expressed alone, however it was detected after co-transfection of both plasmids. The anti-Flag antibody immunoprecipitated pfFasL when it was expressed alone or along with the sFasL plasmid and co-precipitated sFasL after co-expression of both constructs, thereby confirming our hypothesis A densitometric analysis of the immunoblot showed that the association of sFasL increased with the amount of plasmid co-transfected into the cells, for an identical amount of immunoprecipitated pfFasL as quantitated with the ELISA specific for Flag-tagged FasL (Figure 3B). The same experiment was performed with the TCR-pfFasL chimera (Figure 3C). Identical results were obtained, as a small amount of sFasL could be immunoprecipitated with the anti-TCRδ5 antibody only when it was co-expressed together with the chimeric protein.

To eliminate the possibility that the association we observed occurred after the secretion of the proteins, and was merely caused by non specific interactions between the two proteins, we performed the following experiment. The pfFasL, the HLA-pfFasL chimeric proteins and sFasL were expressed separately. The culture supernatant containing the chimera was then mixed with the sFasL supernatant or culture medium, and incubated 24 h at 37°C, mimicking the conditions observed in the co-transfection experiments. Thereafter, the chimera and the total FasL content were measured using both the Flag ELISA and the FasL ELISA assays. In contrast to the results obtained upon plasmid co-transfection (see Figure 2), the anti-FasL tracing antibody of the Flag ELISA did not detect a higher amount of the chimera upon mixing with sFasL (Figure S2 panel A). To confirm the absence of non specific interaction, we performed the same co-immunoprecipitation experiments from these mixed supernatants, for the pfFasL chimera, as those reported in Figure 3A for the co-transfection experiment. We could not detect any association between sFasL and pfFasL in mixed supernatants (Figure 3D). Therefore, we concluded that sFasL physically associated to the chimeric proteins before the release in the supernatant, strongly suggesting that this will occur only upon co-transfection of both plasmids.

### Enhancing the cytotoxic activity of the pfFasL-derived chimeras with sFasL

**Figure 4:**
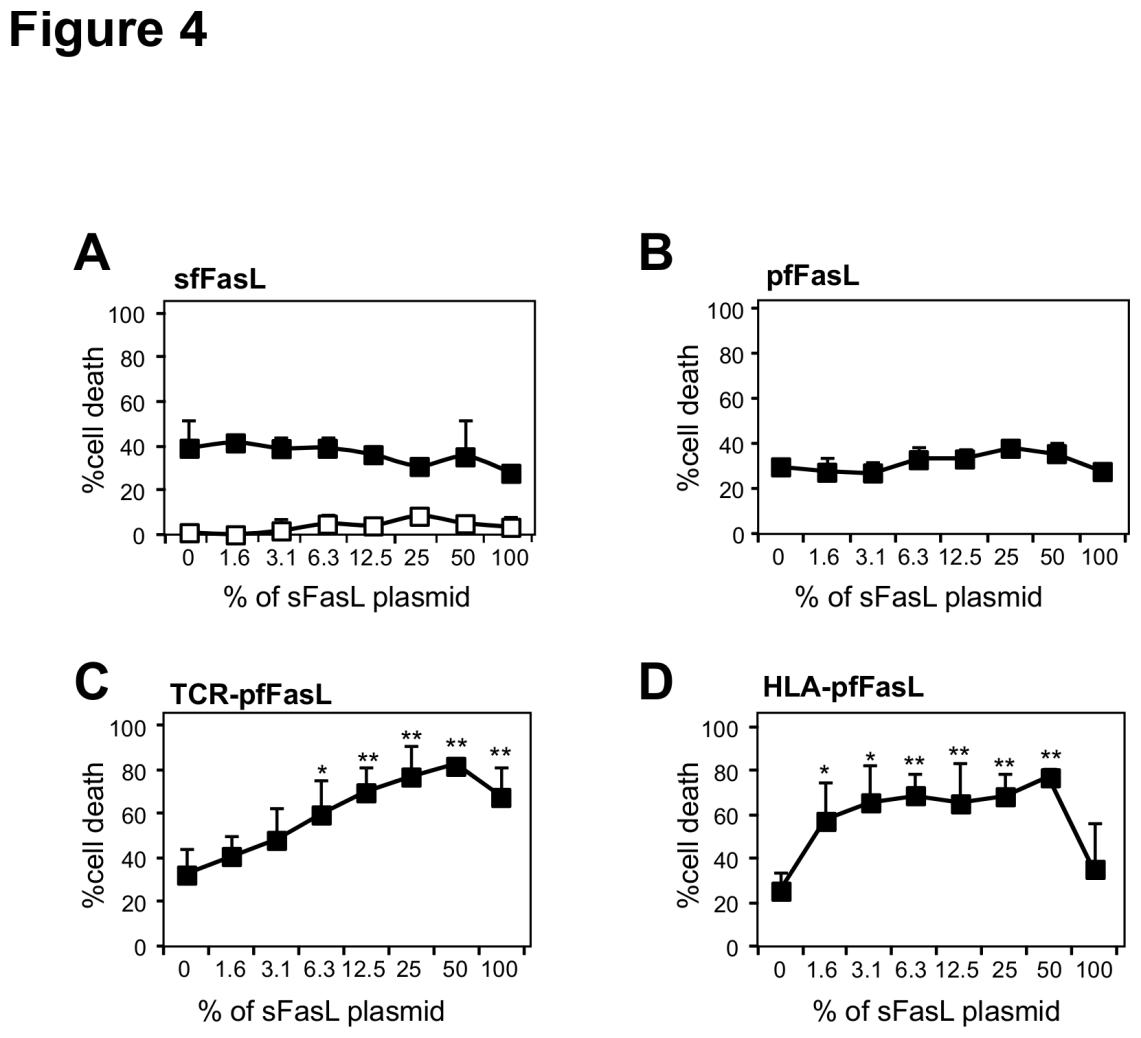
Effect of sFasL on the cytotoxic activity of the Flag-tagged FasL chimeras. The FasL-derived proteins sfFasL (Panel A), pfFasL (Panel B), TCR-pfFasL (Panel C) and HLA-pfFasL (Panel D) were expressed alone or upon co-transfection with the indicated percentage of the plasmid encoding sFasL. A fixed concentration triggering 25 to 40% of cell death (1.9 ng/ml for sfFasL, 0.6 ng/ml for pfFasL, 0.7 ng/ml for HLA-pfFasL and 2.2 ng/ml for TCR-pfFasL), for the FasL-derived protein quantitated with the ELISA specific for Flag-tagged FasL, was incubated with the Fas-sensitive Jurkat cells. For the sfFasL construct, the filled squares and the empty squares depict the cytotoxicity of sfFasL in the presence and absence of the cross-linking anti-Flag antibody at 0.5 µg/ml), respectively. Cytotoxicity was estimated by a measure of the remaining viable cells using the MTT assay. Are presented the mean +/- sd of four independent transfection experiments. * 0.01≤p≤0.05; ** p≤0.01.

We analyzed the effect of sFasL on the capacity of the FasL-derived chimeric proteins to trigger apoptosis of the Fas-sensitive Jurkat cell line (Figure 4). We chose a concentration of the Flag-tagged proteins produced in the absence of added sFasL plasmid, which triggered a weak cytotoxicity, in the 20 to 30% range of cell death. An identical concentration of the Flag-tagged proteins produced in the presence of the sFasL plasmid, as estimated with the ELISA specific for the Flag-tagged constructs, was incubated with the target cells for each of the various sFasL plasmid ratios assayed. Differences in cell cytotoxicity would therefore reflect a change in the intrinsic ability of the co-expressed proteins to trigger cell death, due to the presence of sFasL. The presence of sFasL did not modify the ability of sfFasL cross-linked with the anti-Flag antibody to kill Jurkat cells, over the whole range of plasmid ratios that was tested (Figure 4A). Similar results were obtained for pfFasL, whose cytotoxic activity was not modified in the presence of sFasL (Figure 4B). Interestingly, the TCR-pfFasL and HLA-pfFasL both displayed a 3 to 4 fold improved cytotoxicity in the presence of sFasL plasmid at the optimal molar ratio of 50% (Figure 4C and 4D, respectively). At higher ratios of sFasL plasmid, the gain in cytotoxic activity tended to decrease for TCR-pfFasL and HLA-pfFasL, an effect which was not observed for pfFasL. The MTT cell viability assay was performed with the separately produced pfFasL and HLA-pfFasL, which were mixed with sFasL followed by a 24 h incubation at 37°C. No change in cytotoxic activity was detected for any of the two chimeras (Figure S2, panel B).

### Incorporation of sFasL does not hinder cell targeting of the FasL chimera

**Figure 5:**
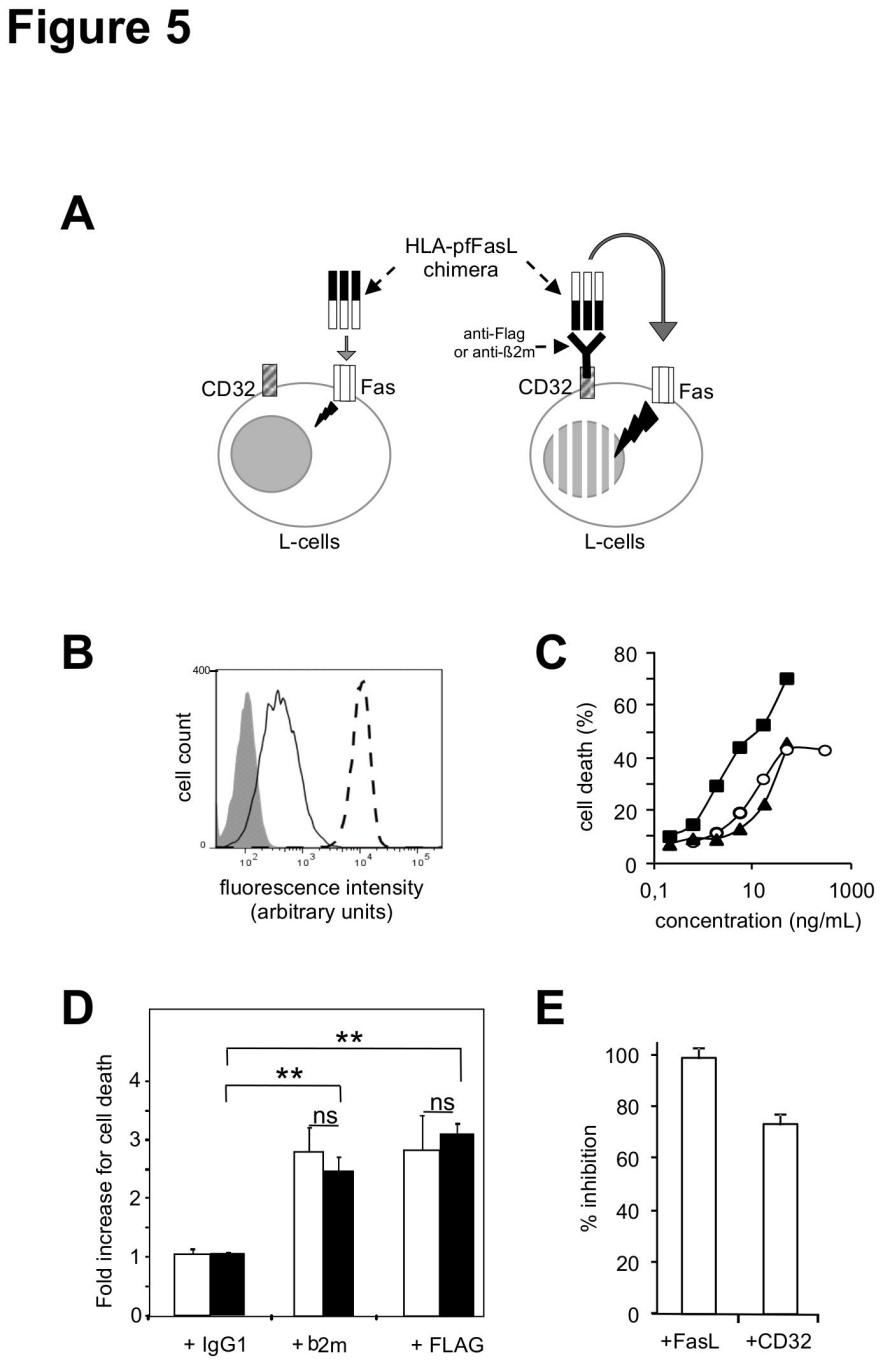
Effect of sFasL on cell targeting of the FasL-containing chimeras. Panel A : Schematic description of the experimental model used. The chimera is enriched at the surface of the CD32-expressing L-cells via its HLA targeting module and an anti-HLA monoclonal antibody. Panel B: murine Fas (continuous line), human CD32 (dashed line) and IgG1 isotype-matched control (shaded histogram) staining of the CD32+ L-cell transfectant. Living cells were gated on the basis of the morphological parameters. Panel C : Fas sensitivity of the CD32+ L-cell transfectant to the indicated concentrations of the anti-Fas JO-2 antibody (circles), the HLA-pfFasL chimera expressed alone (triangle) or in the presence of 25% of the sFasL plasmid (squares), in the MTT viability assay. Panel D : The CD32+ L-cells were incubated with the HLA-pfFasL chimera produced in the presence (black bars) or in the absence (white bars) of 25% of the sFasL plasmid, together with the indicated irrelevant IgG1 isotype-matched, anti-beta-2 microglobulin or anti-Flag antibodies. The concentrations of the chimera that triggered 15% of cell death and were at 15 and 0.3 ng/ml in the absence and presence of sFasL, as estimated using the ELISA specific for the Flag-tagged FasL. Cytotoxicity was measured with the propidium iodide assay and normalized to the effect of the chimera in the absence of antibody. Are presented the mean +/- sd of three independent experiments. Panel E: reversal in the presence of the blocking anti-FasL and anti-CD32 antibodies, of the cytotoxic effect of the immune complexes between the anti-Flag antibody and HLA-pfFasL co-expressed with sFasL. Are presented the mean +/- sd of three independent experiments. ns : non significant ; ** p≤0.02.

As the chimeric pfFasL-derived proteins were designed to target cells to be eliminated through the N-terminal TCR or HLA module, we examined the possibility that despite an enhancement of chimera production and cytotoxic activity, the association of sFasL to the chimeric complex could modify the cell targeting potential of the chimeras. To investigate this possibility, we analyzed the ability of the HLA-pfFasL chimeric protein to target Fas-sensitive cells in a specific manner. For that purpose, we designed the experiment depicted in Figure 5A. We used murine fibroblastic L-cells stably producing the human IgG Fc receptor CD32 and spontaneously expressing murine Fas (Figure 5B). These cells are sensitive to apoptosis induced by the agonistic anti-murine Fas JO-2 antibody and human FasL (Figure 5C), although less than the Jurkat cell line. In order to mimic the targeting effect mediated by the chimera, we pre-incubated the HLA-pfFasL chimera with an anti-Flag or an anti-beta-2 microglobulin antibody, to generate immune complexes, or with an isotype-matched irrelevant monoclonal antibody as a negative control. We used concentrations of the HLA-pfFasL chimera in the presence or absence of sFasL that triggered 15 to 25% of cell death, as measured with the ELISA specific for the Flag-tagged molecule (Figure 5D). The targeting mediated through the anti-beta-2 microglobulin or anti-Flag antibodies via CD32 significantly enhanced by 2.5 fold the cytotoxic activity of the chimeric molecule expressed alone. In the presence of sFasL, the CD32 targeting was fully maintained, as the gain in activity was identical to what was obtained with the chimera expressed without sFasL. The dependency of the cytotoxic effect measured on the L-cells toward Fas and CD32 was verified by its abrogation in the presence of neutralising anti-FasL or blocking anti-CD32 antibodies (Figure 5E). 

## Discussion

In this report, we described the design of polymeric FasL-based chimeric proteins with better heterotopic cellular production and enhanced biological activity. This was achieved by co-expressing the chimeric protein of interest together with sFasL leading to the secretion of heteromeric complexes associating both molecules. At first glance, this may appear as highly counter-intuitive, as sFasL is known to display only a very weak cytotoxic activity, which was confirmed in our experiments as it was at least 5000 times less active than pfFasL. Also, as a weak agonist, sFasL inhibits Fas-mediated apoptosis triggered by highly active FasL polymers or agonistic anti-Fas antibodies. However, this occurs only in the presence of very high concentrations of sFasL, in the range of 300-1000 fold that of the active pfFasL-derived chimera (Figure S3). Nevertheless, we indeed observed that the association of sFasL with the FasL-derived chimeras increased both their recovery in the culture supernatant and their proapoptotic functional activity.

The gain in net chimeric protein production seemed to depend on the complexity and/or the size of the FasL-based monomer, which by itself already greatly influenced the level of production that could be reached in the absence of the sFasL plasmid. We did not observe any effect of co-expressed sFasL on the net production of trimeric sfFasL, likely because it is already produced at saturating levels when expressed alone in the optimized experimental conditions used. For pfFasL, which is polymeric [21], the production of this chimera is enhanced by up to 10 fold in the presence of sFasL. For more complex FasL-based chimeras, such as HLA-pfFasL and TCR-pfFasL, production is also improved, but to a lower 2 to 5 fold range. When these chimeras are produced alone, they are secreted at much lower levels than the smaller sfFasL and pfFasL forms, leading us to conclude that significantly improving their production is indeed possible but that intrinsic constraints, such as the size of the monomer, of the final polymer or of both, will nevertheless auto-limit the capacity of the cell to produce and/or release them. The phenomenon we describe in the present report was not limited to a specific chimeric construct, as we observed it with three different ones. It was not dependent on the cell production system used, as the TCR and HLA chimeras were produced in HEK and COS cells, respectively. In experiments not shown, we obtained similar results for the pfFasL alone or in combination with sFasL in a very different context, i.e. stable production following transduction of HEK cells with two lentiviral constructs each encoding one FasL-derived molecule (Figure S4). Our results also showed that the gain in protein production reached a maximum before decreasing when the proportion of sFasL becomes too important, as was observed for sfFasL, pfFasL, HLA-pfFasL and TCR-pfFasL. This suggests that an overwhelming production of sFasL might divert the cellular machinery from manufacturing of the HLA-pfFasL and TCR-pfFasL chimeras.

The gain in function we observed was also dependent on the complexity of the FasL-based monomer, with some differences when compared to the improvement in production. For sfFasL, no cytotoxic function appeared in the presence of co-transfected sFasL, consistent with the fact that sFasL is not expected to alter sfFasL polymerisation, which by itself is trimeric as sFasL is [9]. No gain in function was noticed either in the presence of the cross-linking anti-Flag antibody, suggesting that the spatial intrinsic organization of the sfFasL+anti-Flag antibody is close to its functional optimum, and therefore cannot be improved further with sFasL. This is confirmed with the pfFasL chimera, as no improvement in cytotoxic efficiency was observed in the presence of sFasL, although we reported a strong increase in the amount of protein produced. The discrepancy between these two criteria also suggests that the spatial organization of the pfFasL chimera is already optimal in the absence of sFasL, and that only its intracellular processing or release can be optimized. For the HLA-pfFasL and TCR-pfFasL species, which were produced in much lower amounts, the cytotoxic activity was significantly enhanced, in addition to an improvement in cell production, in the presence of sFasL. Therefore, the gain occurs at both steps. However, although the overall increase might be considered as modest for each step taken individually, this may be explained by the structure of the chimeras we attempted to produce. Indeed, a γ4δ5 TCR is a noncovalently-linked heterodimeric protein with a natural propensity for the two chains to interact with each other into a stable dimer. In experiments not shown, we noticed that none of the two chimeric chains was produced alone, in the absence of the co-transfection of its partner, suggesting that the pre-association of the TCR chains is a pre-requisite to the release of the TCR-pfFasL chimera. Therefore, such a polymeric chimera is intrinsically structurally complex, which may explain why the spontaneous production level is low, and also why it cannot be drastically improved. An alternative would be to generate a single chain construct, on the model of what has been done for a TCR of the alpha-beta type [33]. In the case of HLA-pfFasL, the construct we used consisted in a single chain beta-2 microglobulin HLA fusion construct, secondly attached to the pfFasL moiety, so the HLA targeting module was already a complex molecule on its own. In addition, HLA stability is highly dependent on the presence of a peptide into the peptide-binding groove, which may also impinge on the overall stability of the chimera. Our results also showed that the gain in cytotoxic activity for HLA-pfFasL and TCR-pfFasL due to sFasL, reached a maximum before decreasing in the presence of the highest proportions of sFasL. This phenomenon could be explained by the inhibitory effect of sFasL at very high concentrations, on the cytotoxicity triggered by active Fas agonists, as the sFasL/chimera ratio reached the 300 to 1000 fold level required to uncover this effect (Figure S3). This effect can be evidenced for TCR-pfFasL and HLA-pfFasL but not for pfFasL, because the production of these chimeras drastically decreases in the presence of high amounts of the sFasL plasmid (Figure 2C and 2D), while sFasL production still increases. The threshold required to uncover sFasL inhibitory activiy is then reached for TCR-pfFasL and HLA-pfFasL, which is not the case for pfFasL (Figure 2B).

We observed that sFasL physically interacted with the chimeric pfFasL-derived proteins. This is not a passive phenomenon, as it cannot be obtained if the proteins are produced separately, before being mixed together. This strongly suggests a tight association of both kinds of components into a heteropolymer at the time the chimera is built. This could explain the increase in production through a possible facilitation of intracellular protein maturation until it is released in the extracellular milieu. To analyze and compare the composition of the chimeras produced in the presence and absence of sFasL, we performed preliminary gel filtration experiments. We observed a decrease in the size of the pfFasL polymer when expressed in the presence of sFasL, but we were not able to separate to homogeneity the individual compounds that coexisted in the cell culture supernatant (results not shown).

Our results suggest that this design could improve the efficacy of cell type-selective chimeras, as we describe that the cytotoxicity of the HLA-pfFasL towards Fas-sensitive cells is maintained in a cellular model where the chimera is tethered onto the surface of a presenting cell via an anti-beta2 microglobulin or an anti-Flag antibody. Then, the approach we describe here suggests that the design of FasL-derived chimeras associating two different cell-targeting modules is possible, with possibly a synergy as the coexpression of two different monomers could lead to a copolymer with a higher activity than each constitutive compound.

## Supporting Information

Figure S1 **Effect of plasmid amount on production of the FasL-derived chimeras.**
An increasing amount of plasmid encoding pfFasL, HLA-pfFasL or TCR-pfFasL was transfected into mammalian COS or HEK cells. The proteins excreted in culture supernatant were quantified using the Flag ELISA. Shown is one of two independent experiments.
(TIF)

Figure S2 **measurement and activity of the pfFasL and HLA-pfFasL chimeric proteins after mixing with sFasL produced separately.**
HLA-pfFasL and pfFasL proteins produced by COS cells were mixed with sFasL (1: 1 volume of culture supernatant) produced separately, and incubated for 24 h at 37°C. As a negative control, each of the chimeras was mixed with culture medium. Panel A: The FasL proteins were quantified using an ELISA specific for total sFasL (upper panel) and for Flag-tagged sFasL proteins (lower panel). Panel B: the cytotoxic activity of the chimeras mixed with medium (squares) or sFasL (circles) was measured with the MTT viability assay at the indicated concentrations of the chimeras as measured with the Flag ELISA.
(TIF)

Figure S3 **Blocking by sFasL of the cytotoxic effect of the FasL-derived chimeras.**
The sFasL protein was pre-incubated with Jurkat cells at the indicated concentrations. A fixed concentration of pfFasL (6 ng/ml), sfFasL (50 ng/ml), TCR-pfFasL (50 ng/ml) or HLA-pfFasL (9 ng/ml), determined as being able to trigger a subtotal cell death among Jurkat cells (condition without added sFasL), was then added. Cell viability was measured 18 h later with a MTT assay. The anti-Flag M2 antibody (1 µg/ml) was added to render sfFasL cytotoxic. Are presented the mean +/- sd of four independent transfection experiments. * p<0.02.
(TIF)

Figure S4 **Effect of sFasL on pfFasL production by HEK cells upon lentiviral co-transduction.**
HEK cells were transduced with a vector encoding sFasL and Green Fluorescent Protein. The resulting HEK-sFasL+ cell line and the wild-type HEK were transduced with a vector encoding pfFasL and Tomato. Cells were FACS-sorted for weak (HEK-pfFasL+) or strong (HEK-pfFasL++) Tomato expression. Secreted pfFasL was quantified with the Flag ELISA.
(TIF)

## References

[1] Bodmer JL, Schneider P, Tschopp J (2002) The molecular architecture of the TNF superfamily. Trends Biochem Sci 27: 19-26. doi:10.1016/S0968-0004(01)01995-8. PubMed: 11796220. 11796220 10.1016/s0968-0004(01)01995-8

[2] Krueger A, Fas SC, Baumann S, Krammer PH (2003) The role of CD95 in the regulation of peripheral T-cell apoptosis. Immunol Rev 193: 58-69. doi:10.1034/j.1600-065X.2003.00047.x. PubMed: 12752671. 12752671 10.1034/j.1600-065x.2003.00047.x

[3] Kayagaki N, Kawasaki A, Ebata T, Ohmoto H, Ikeda S et al. (1995) Metalloproteinase-mediated release of human Fas ligand. J Exp Med 182: 1777-1783. doi:10.1084/jem.182.6.1777. PubMed: 7500022. 7500022 10.1084/jem.182.6.1777 PMC2192231

[4] Mariani SM, Matiba B, Bäumler C, Krammer PH (1995) Regulation of cell surface APO-1/Fas (CD95) ligand expression by metalloproteases. Eur J Immunol 25: 2303-2307. doi:10.1002/eji.1830250828. PubMed: 7545118. 7545118 10.1002/eji.1830250828

[5] Suda T, Hashimoto H, Tanaka M, Ochi T, Nagata S (1997) Membrane Fas ligand kills human peripheral blood T lymphocytes, and soluble Fas ligand blocks the killing. J Exp Med 186: 2045-2050. doi:10.1084/jem.186.12.2045. PubMed: 9396774. 9396774 10.1084/jem.186.12.2045 PMC2199173

[6] Schneider P, Holler N, Bodmer JL, Hahne M, Frei K et al. (1998) Conversion of membrane-bound Fas(CD95) ligand to its soluble form is associated with downregulation of its proapoptotic activity and loss of liver toxicity. J Exp Med 187: 1205-1213. doi:10.1084/jem.187.8.1205. PubMed: 9547332. 9547332 10.1084/jem.187.8.1205 PMC2212219

[7] Tanaka M, Itai T, Adachi M, Nagata S (1998) Downregulation of Fas ligand by shedding. Nat Med 4: 31-36. doi:10.1038/nm0198-031. PubMed: 9427603. 9427603 10.1038/nm0198-031

[8] Sieg S, Smith D, Kaplan D (1999) Differential activity of soluble versus cellular Fas ligand: regulation by an accessory molecule. Cell Immunol 195: 89-95. doi:10.1006/cimm.1999.1530. PubMed: 10448008. 10448008 10.1006/cimm.1999.1530

[9] Holler N, Tardivel A, Kovacsovics-Bankowski M, Hertig S, Gaide O et al. (2003) Two adjacent trimeric Fas ligands are required for Fas signaling and formation of a death-inducing signaling complex. Mol Cell Biol 23: 1428-1440. doi:10.1128/MCB.23.4.1428-1440.2003. PubMed: 12556501. 12556501 10.1128/MCB.23.4.1428-1440.2003 PMC141146

[10] Ogasawara J, Watanabe-Fukunaga R, Adachi M, Matsuzawa A, Kasugai T et al. (1993) Lethal effect of the anti-Fas antibody in mice. Nature 364: 806-809. doi:10.1038/364806a0. PubMed: 7689176. 7689176 10.1038/364806a0

[11] Saito-Yabe M, Yoshigae Y, Takasaki W, Kurihara A, Ikeda T et al. (2009) Highly frequent anti-idiotype antibody in cynomolgus monkeys developed against mouse-derived regions of anti-Fas antibody humanized by complementarity determining region grafting. Br J Pharmacol 158: 548-557. doi:10.1111/j.1476-5381.2009.00326.x. PubMed: 19645714. 19645714 10.1111/j.1476-5381.2009.00326.x PMC2757695

[12] de Bruyn M, Bremer E, Helfrich W (2011) Antibody-based fusion proteins to target death receptors in cancer. Cancer Lett, 332: 175–83. PubMed: 21215513. 21215513 10.1016/j.canlet.2010.11.006

[13] Villa-Morales M, Fernandez-Piqueras J (2012) Targeting the Fas/FasL signaling pathway in cancer therapy. Expert Opin Ther Targets 16: 85-101. doi:10.1517/14728222.2011.628937. PubMed: 22239437. 22239437 10.1517/14728222.2011.628937

[14] Yolcu ES, Askenasy N, Singh NP, Cherradi SE, Shirwan H (2002) Cell membrane modification for rapid display of proteins as a novel means of immunomodulation: FasL-decorated cells prevent islet graft rejection. Immunity 17: 795-808. doi:10.1016/S1074-7613(02)00482-X. PubMed: 12479825. 12479825 10.1016/s1074-7613(02)00482-x

[15] Huang JH, Tykocinski ML (2001) CTLA-4-Fas ligand functions as a trans signal converter protein in bridging antigen-presenting cells and T cells. Int Immunol 13: 529-539. doi:10.1093/intimm/13.4.529. PubMed: 11282992. 11282992 10.1093/intimm/13.4.529

[16] Elhalel MD, Huang JH, Schmidt W, Rachmilewitz J, Tykocinski ML (2003) CTLA-4. FasL induces alloantigen-specific hyporesponsiveness. J Immunol 170: 5842-5850. PubMed: 12794109. 12794109 10.4049/jimmunol.170.12.5842

[17] Samel D, Muller D, Gerspach J, Assohou-Luty C, Sass G et al. (2003) Generation of a FasL-based proapoptotic fusion protein devoid of systemic toxicity due to cell-surface antigen-restricted Activation. J Biol Chem 278: 32077-32082. doi:10.1074/jbc.M304866200. PubMed: 12773535. 12773535 10.1074/jbc.M304866200

[18] Bremer E, ten Cate B, Samplonius DF, Mueller N, Wajant H et al. (2008) Superior activity of fusion protein scFvRit:sFasL over cotreatment with rituximab and Fas agonists. Cancer Res 68: 597-604. doi:10.1158/0008-5472.CAN-07-5171. PubMed: 18199557. 18199557 10.1158/0008-5472.CAN-07-5171

[19] Zhang W, Wang F, Wang B, Zhang J, Yu JY (2012) Intraarticular gene delivery of CTLA4-FasL suppresses experimental arthritis. Int Immunol 24: 379-388. doi:10.1093/intimm/dxs041. PubMed: 22354915. 22354915 10.1093/intimm/dxs041

[20] Eisele G, Roth P, Hasenbach K, Aulwurm S, Wolpert F et al. (2011) APO010, a synthetic hexameric CD95 ligand, induces human glioma cell death in vitro and in vivo. Neuro Oncol 13: 155-164. doi:10.1093/neuonc/noq176. PubMed: 21183510. 21183510 10.1093/neuonc/noq176 PMC3064626

[21] Daburon S, Devaud C, Costet P, Morello A, Garrigue-Antar L et al. (2013) Functional characterization of a chimeric soluble Fas ligand polymer with in vivo anti-tumor activity. PLOS ONE 8: e54000. doi:10.1371/journal.pone.0054000. PubMed: 23326557. 23326557 10.1371/journal.pone.0054000 PMC3541234

[22] Willcox CR, Pitard V, Netzer S, Couzi L, Salim M et al. (2012) Cytomegalovirus and tumor stress-surveillance by human γδ T cell receptor binding to Endothelial Protein C Receptor. Nat Immunol 13: 872-879. doi:10.1038/ni.2394. PubMed: 22885985. 22885985 10.1038/ni.2394

[23] Banchereau J, de Paoli P, Vallé A, Garcia E, Rousset F (1991) Long-term human B cell lines dependent on interleukin-4 and antibody to CD40. Science 251: 70-72. doi:10.1126/science.1702555. PubMed: 1702555. 1702555 10.1126/science.1702555

[24] Gluzman Y (1981) SV40-transformed simian cells support the replication of early SV40 mutants. Cell 23: 175-182. doi:10.1016/0092-8674(81)90282-8. PubMed: 6260373. 6260373 10.1016/0092-8674(81)90282-8

[25] Sena-Esteves M, Saeki Y, Camp SM, Chiocca EA, Breakefield XO (1999) Single-step conversion of cells to retrovirus vector producers with herpes simplex virus-Epstein-Barr virus hybrid amplicons. J Virol 73: 10426-10439. PubMed: 10559361. 10559361 10.1128/jvi.73.12.10426-10439.1999 PMC113098

[26] Vivier E, Rochet N, Ackerly M, Petrini J, Levine H et al. (1992) Signaling function of reconstituted CD16: zeta: gamma receptor complex isoforms. Int Immunol 4: 1313-1323. doi:10.1093/intimm/4.11.1313. PubMed: 1472481. 1472481 10.1093/intimm/4.11.1313

[27] Legembre P, Moreau P, Daburon S, Moreau JF, Taupin JL (2002) Potentiation of Fas-mediated apoptosis by an engineered glycosylphosphatidylinositol-linked Fas. Cell Death Differ 9: 329-339. doi:10.1038/sj.cdd.4400960. PubMed: 11859415. 11859415 10.1038/sj.cdd.4400960

[28] Taupin JL, Gualde N, Moreau JF (1997) A monoclonal antibody based elisa for quantitation of human leukaemia inhibitory factor. Cytokine 9: 112-118. doi:10.1006/cyto.1996.0144. PubMed: 9071562. 9071562 10.1006/cyto.1996.0144

[29] Kaufman RJ, Davies MV, Wasley LC, Michnick D (1991) Improved vectors for stable expression of foreign genes in mammalian cells by use of the untranslated leader sequence from EMC virus. Nucleic Acids Res 19: 4485-4490. doi:10.1093/nar/19.16.4485. PubMed: 1653417. 1653417 10.1093/nar/19.16.4485 PMC328638

[30] Jordan M, Wurm F (2004) Transfection of adherent and suspended cells by calcium phosphate. Methods 33: 136-143. doi:10.1016/j.ymeth.2003.11.011. PubMed: 15121168. 15121168 10.1016/j.ymeth.2003.11.011

[31] Schneider P, Bodmer JL, Holler N, Mattmann C, Scuderi P et al. (1997) Characterization of Fas (Apo-1, CD95)-Fas ligand interaction. J Biol Chem 272: 18827-18833. doi:10.1074/jbc.272.30.18827. PubMed: 9228058. 9228058 10.1074/jbc.272.30.18827

[32] Jordan M, Schallhorn A, Wurm FM (1996) Transfecting mammalian cells: optimization of critical parameters affecting calcium-phosphate precipitate formation. Nucleic Acids Res 24: 596-601. doi:10.1093/nar/24.4.596. PubMed: 8604299. 8604299 10.1093/nar/24.4.596 PMC145683

[33] Belmont HJ, Price-Schiavi S, Liu B, Card KF, Lee HI et al. (2006) Potent antitumor activity of a tumor-specific soluble TCR/IL-2 fusion protein. Clin Immunol 121: 29-39. doi:10.1016/j.clim.2006.05.005. PubMed: 16807113. 16807113 10.1016/j.clim.2006.05.005

