# Supplementary figures and images for: Enhancing Production and Cytotoxic Activity of Polymeric Soluble FasL-Based Chimeric Proteins by Concomitant Expression of Soluble FasL
Source: PLoS One. 2013 Aug 26;8(8):e73375. doi: 10.1371/journal.pone.0073375 (PMC3753252; doi:10.1371/journal.pone.0073375)

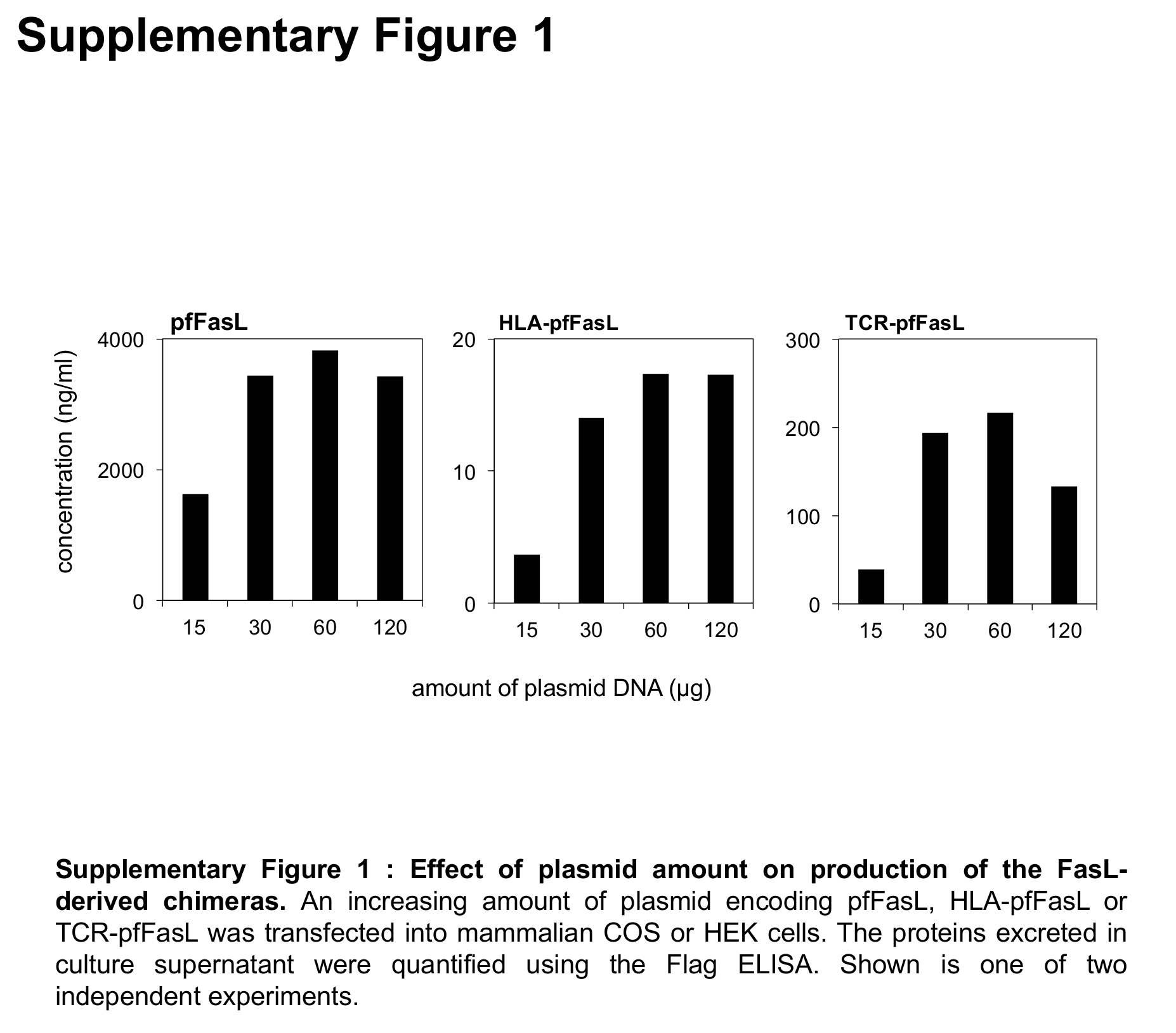

Supplement: Figure S1 — Effect of plasmid amount on production of the FasL-derived chimeras. An increasing amount of plasmid encoding pfFasL, HLA-pfFasL or TCR-pfFasL was transfected into mammalian COS or HEK cells. The proteins excreted in culture supernatant were quantified using the Flag ELISA. Shown is one of two independent experiments. (TIF) [file pone.0073375.s001.tif]

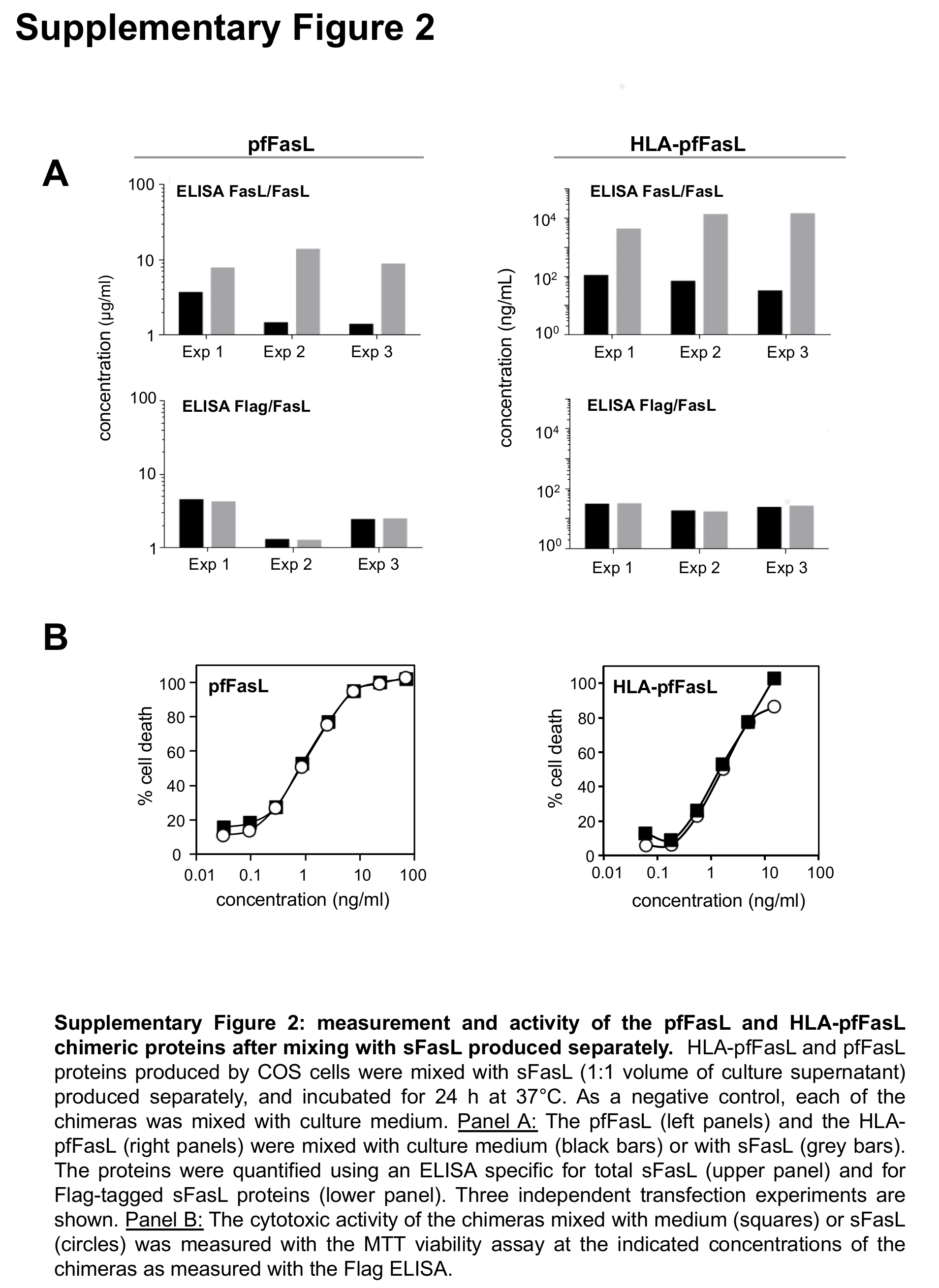

Supplement: Figure S2 — measurement and activity of the pfFasL and HLA-pfFasL chimeric proteins after mixing with sFasL produced separately. HLA-pfFasL and pfFasL proteins produced by COS cells were mixed with sFasL (1: 1 volume of culture supernatant) produced separately, and incubated for 24 h at 37°C. As a negative control, each of the chimeras was mixed with culture medium. Panel A: The FasL proteins were quantified using an ELISA specific for total sFasL (upper panel) and for Flag-tagged sFasL proteins (lower panel). Panel B: the cytotoxic activity of the chimeras mixed with medium (squares) or sFasL (circles) was measured with the MTT viability assay at the indicated concentrations of the chimeras as measured with the Flag ELISA. (TIF) [file pone.0073375.s002.tif]

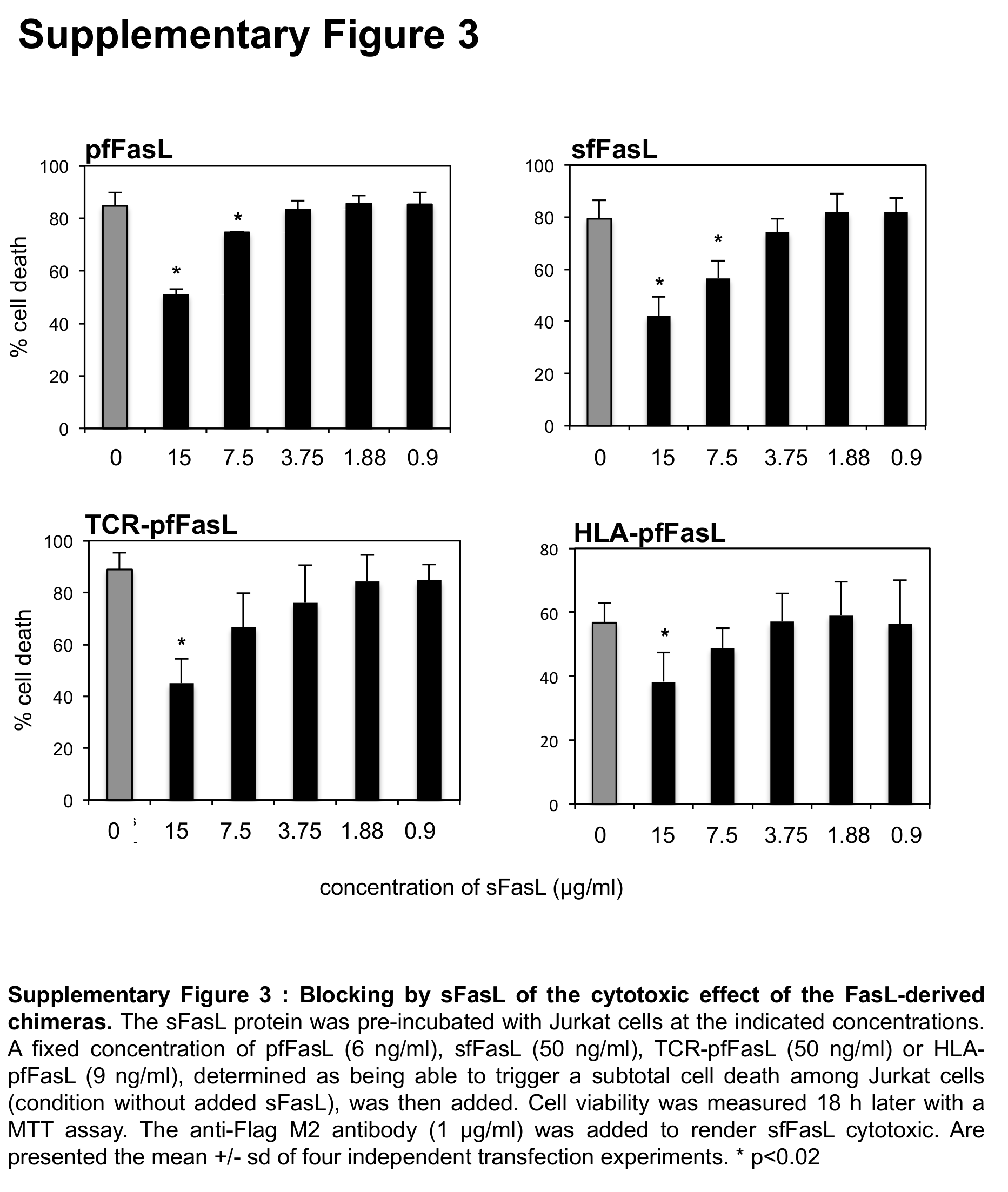

Supplement: Figure S3 — Blocking by sFasL of the cytotoxic effect of the FasL-derived chimeras. The sFasL protein was pre-incubated with Jurkat cells at the indicated concentrations. A fixed concentration of pfFasL (6 ng/ml), sfFasL (50 ng/ml), TCR-pfFasL (50 ng/ml) or HLA-pfFasL (9 ng/ml), determined as being able to trigger a subtotal cell death among Jurkat cells (condition without added sFasL), was then added. Cell viability was measured 18 h later with a MTT assay. The anti-Flag M2 antibody (1 µg/ml) was added to render sfFasL cytotoxic. Are presented the mean +/- sd of four independent transfection experiments. * p<0.02. (TIF) [file pone.0073375.s003.tif]

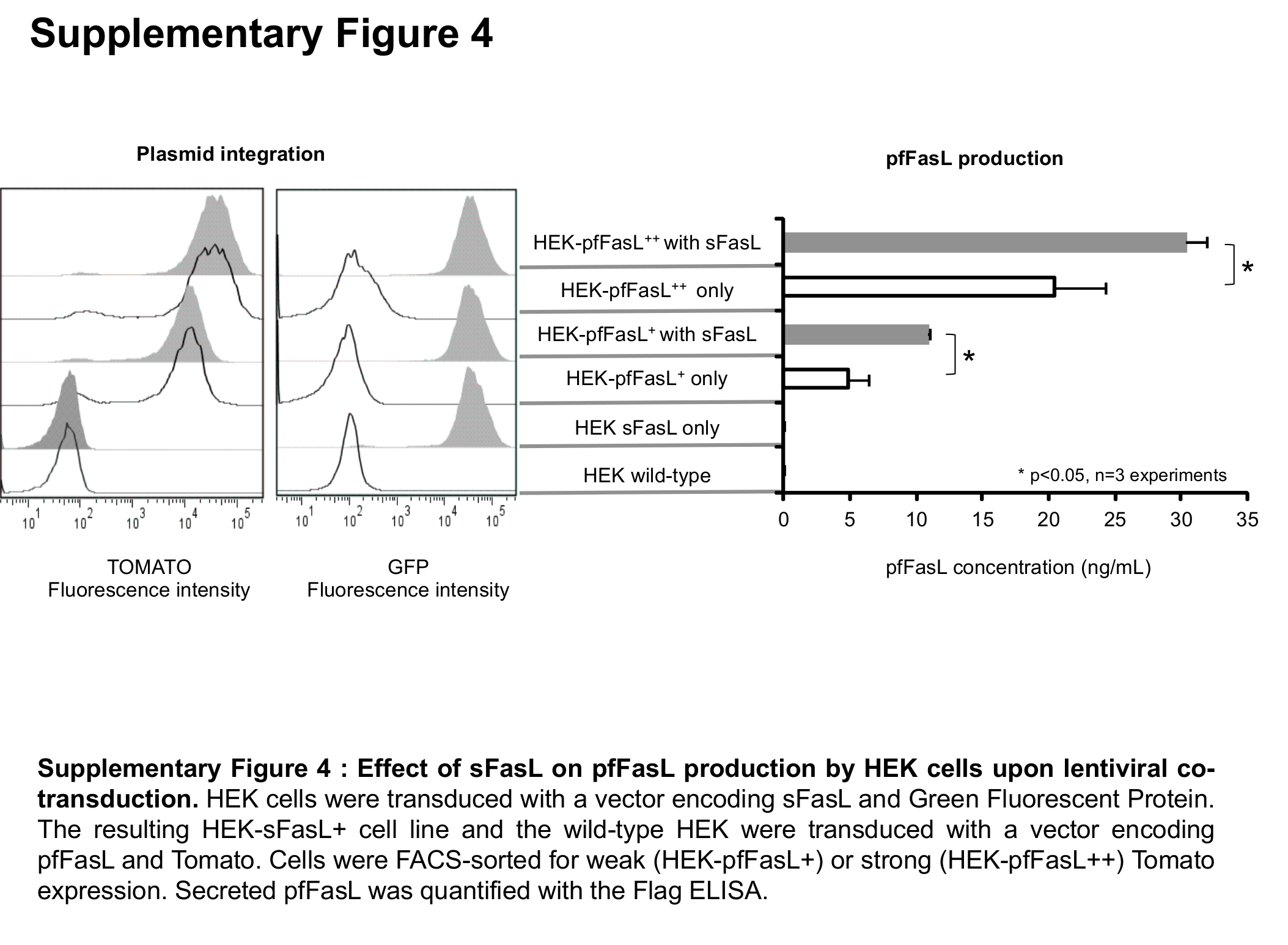

Supplement: Figure S4 — Effect of sFasL on pfFasL production by HEK cells upon lentiviral co-transduction. HEK cells were transduced with a vector encoding sFasL and Green Fluorescent Protein. The resulting HEK-sFasL+ cell line and the wild-type HEK were transduced with a vector encoding pfFasL and Tomato. Cells were FACS-sorted for weak (HEK-pfFasL+) or strong (HEK-pfFasL++) Tomato expression. Secreted pfFasL was quantified with the Flag ELISA. (TIF) [file pone.0073375.s004.tif]
